# FOXO6 inhibits SMURF2‐mediated ubiquitination and degradation of NR4A1 to promote retinal pigment epithelial cell ferroptosis in diabetic retinopathy

**DOI:** 10.1002/ccs3.70108

**Published:** 2026-08-26

**Authors:** Qiao Chen, Bo Su, Ke Xu, Liang Li, Changzheng Chen

**Affiliations:** ^1^ Department of Ophthalmology Renmin Hospital of Wuhan University Wuhan Hubei China; ^2^ Department of Pathology Medical School of Yangtze University Jingzhou Hubei China; ^3^ Department of Ophthalmology Jingzhou Hospital Affiliated to Yangtze University Jingzhou Hubei China

**Keywords:** diabetic retinopathy, ferroptosis, Forkhead box O6, nuclear receptor subfamily 4 group A member 1, SMAD‐specific E3 ubiquitin protein ligase 2, ubiquitination

## Abstract

Diabetic retinopathy (DR) is the main cause of vision loss, with retinal pigment epithelial (RPE) cell dysfunction as a key contributor. Ferroptosis is implicated in RPE injury under hyperglycemia; however, its upstream regulatory mechanisms remain unclear. High glucose (HG)‐induced human RPE ARPE‐19 cells and a diabetic mouse model were used. Ferroptosis was assessed through biochemical assays, Western blot, fluorescence probes, and histological staining. Protein interactions and transcriptional regulation were examined using Co‐IP, glutathione S‐transferase pull‐down, ChIP, and luciferase reporter assays. Immunofluorescence staining was used as key indicator expression in retinal tissues. Nuclear receptor 4A1 (NR4A1) expression was upregulated under HG‐induced ARPE‐19 cells. NR4A1 knockdown alleviated HG‐induced iron accumulation, lipid peroxidation, and ferroptosis marker changes. Mechanistically, SMAD‐specific E3 ubiquitin protein ligase 2 (SMURF2) interacted with NR4A1 and promoted its ubiquitination and degradation. Forkhead box O6 (FOXO6) was upregulated by HG and repressed SMURF2 transcription. SMURF2 overexpression mitigated HG‐induced ARPE‐19 ferroptosis and retinal injury in DR mice, an effect reversed by NR4A1 co‐overexpression. SMURF2 knockdown reversed the effect of FOXO6 knockdown on HG‐induced ferroptosis in ARPE‐19 cells and retinal injury in DR mice. The FOXO6–SMURF2–NR4A1 axis critically regulates ferroptosis in RPE cells during DR.

## Graphical abstract

FOXO6 suppresses the transcriptional expression of SMURF2, thereby limiting the ubiquitination and degradation of NR4A1. Stabilized NR4A1 promotes ferroptosis in RPE cells, contributing to the progression of diabetic retinopathy.

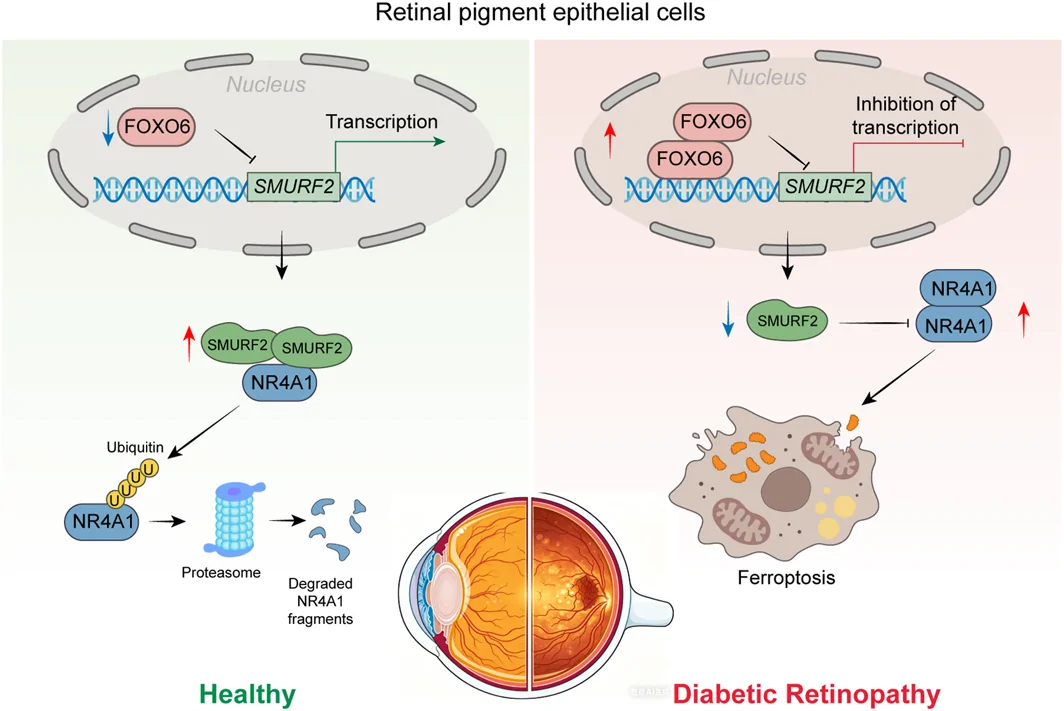

## 1 INTRODUCTION

Diabetic retinopathy (DR) represents a prevalent microvascular disorder arising from chronic diabetes, affecting approximately 35% of diabetic patients.^1^ Persistent hyperglycemia is a major risk factor and contributes to progressive retinal damage, potentially leading to vision impairment or blindness.^2^ Pathologically, DR is characterized by microvascular leakage, inflammation, neovascularization, and neural degeneration.^3^ Despite the availability of anti‐vascular endothelial growth factor (anti‐VEGF) therapies and laser treatments, many patients continue to experience disease progression,^4^ underscoring the need for novel therapeutic targets and deeper mechanistic understanding. The retinal pigment epithelium (RPE), located between the choroidal vasculature and photoreceptor outer segments, plays a critical role in maintaining retinal homeostasis.^5^ Increasing evidence suggests that RPE dysfunction significantly contributes to DR pathogenesis by disrupting oxidative balance, barrier integrity, and immune regulation.^6^ Ferroptosis is an iron‐dependent, lipid peroxidation‐mediated mode of regulated cell death that has been increasingly implicated in RPE cell damage during DR.^7^ This process is triggered by intracellular iron overload and ROS accumulation, leading to membrane damage and cell death.^8^ Notably, pharmacological inhibition of ferroptosis has been shown to alleviate retinal injury in diabetic models.^9^, ^10^ However, the upstream molecular regulators governing RPE ferroptosis in DR remain largely undefined.

Nuclear receptor 4A1 (NR4A1), also known as Nur77, belongs to the orphan nuclear receptor family and participates in a wide range of physiological and pathological processes, including apoptosis, metabolism, and inflammation.^11^ Recent evidence indicates that NR4A1 also plays a role in ferroptosis.^12^, ^13^ Furthermore, NR4A1 expression was elevated in the retina of high‐fat diet‐induced metabolic stress models,^14^ suggesting its involvement in retinal dysfunction. Although NR4A1 has been associated with ferroptosis in various systems, its role in RPE ferroptosis under diabetic conditions remains unclear. Whether suppression of NR4A1 activity can mitigate ferroptosis and RPE degeneration in DR remains to be clarified.

SMAD‐specific E3 ubiquitin protein ligase 2 (SMURF2) is a well‐characterized E3 ligase that modulates the stability of target proteins through ubiquitination.^15^ In RPE cells, SMURF2 has been reported to influence epithelial–mesenchymal transition and barrier permeability in response to high glucose (HG).^6^ However, reports on SMURF2 in DR are limited, and its role in ferroptosis of RPE cells remains unclear. Given its involvement in post‐translational protein regulation, we explored the possibility that SMURF2 interacts with NR4A1 to modulate its stability. Bioinformatic predictions indicated a potential interaction between SMURF2 and NR4A1, suggesting that SMURF2 may facilitate the ubiquitin‐dependent degradation of NR4A1 and thereby attenuate ferroptosis in RPE cells.

Forkhead box O6 (FOXO6) is a member of the FOXO transcription factor family and plays roles in regulating oxidative stress, metabolism, and cell survival.^16^ FOXO6 can function either as a transcriptional activator ^17^ or a repressor,^18^ depending on cellular context. In retinal cells, FOXO6 has been implicated in promoting oxidative stress and apoptosis under high‐glucose conditions.^19^ To identify upstream regulators of SMURF2, we conducted promoter analysis and identified multiple FOXO6 binding motifs in the SMURF2 promoter, suggesting a direct transcriptional relationship. However, the regulatory function of FOXO6 in modulating SMURF2 expression and its impact on RPE ferroptosis remain unexplored.

Based on these findings, we hypothesized that FOXO6 transcriptionally repressed SMURF2, resulting in reduced ubiquitination and degradation of NR4A1, which led to NR4A1 accumulation and promoted ferroptosis in RPE cells, thereby contributing to the progression of DR. Targeting SMURF2 may thus represent a promising strategy to modulate NR4A1‐mediated ferroptosis in the diabetic retina. This study aims to elucidate the regulatory relationships among FOXO6, SMURF2, and NR4A1 and to define their collective role in the ferroptotic pathway underlying DR pathogenesis.

## 2 MATERIALS AND METHODS

### 2.1 Cell culture and treatments

The adult retinal pigment epithelial cell line ARPE‐19 (RRID: CVCL_0145), which was isolated from the in situ retinal pigment epithelium of a 19‐year‐old male, was purchased from ATCC (Cat# CRL‐2302, Manassas, VA, USA, bought time: January 2025). Cell identity was verified via short tandem repeat profiling, and mycoplasma contamination was excluded using PCR and culture assays. Cells were cultured in Dulbecco's Modified Eagle Medium/Nutrient Mixture F‐12 (DMEM/F‐12; Cat# 11320033, Gibco, Thermo Fisher Scientific, Waltham, MA, USA) supplemented with 10% fetal bovine serum (FBS; Cat# 10099141C, Gibco) and 1% penicillin–streptomycin (Cat# 15140122, Gibco) at 37°C in a humidified atmosphere containing 5% CO_2_.

To simulate diabetic conditions, cells were treated with high glucose (HG; 30 mM D‐glucose; Cat# G7021, Sigma‐Aldrich, St. Louis, MO, USA) for 48 h. Control cells were maintained in normal glucose medium containing 5.5 mM D‐glucose. Where indicated, the ferroptosis inhibitor ferrostatin‐1 (Fer‐1; Cat# S7243, Selleck Chemicals, Houston, TX, USA) was added at a final concentration of 1 μM during the last 24 h of HG treatment. For protein stability analysis, cycloheximide (CHX; Cat# C7698, Sigma‐Aldrich) was applied at 100 μg/mL.

### 2.2 Plasmid construction and cell transfection

Short hairpin RNAs (shRNAs) targeting NR4A1, SMURF2, and FOXO6 (sh‐NR4A1, sh‐SMURF2, sh‐FOXO6), along with a nontargeting control (sh‐NC), were cloned into the pGPU6/Neo plasmid vector (GenePharma, Shanghai, China). For overexpression, full‐length human SMURF2 cDNA was inserted into the pcDNA3.1 (+) vector (Invitrogen, Thermo Fisher Scientific), and the corresponding empty vector was used as the overexpression control (oe‐NC). ARPE‐19 cells were seeded at a density of 2 × 10^5^ cells per well in 6‐well plates and incubated overnight. The next day, cells were transfected with 2 μg plasmid DNA per well using Lipofectamine 3000 (Cat# L3000015, Thermo Fisher Scientific) following the manufacturer's protocol. Cells were maintained for 48 h post‐transfection before subsequent treatments or analyses.

### 2.3 RNA extraction and quantitative real‐time PCR (RT‐qPCR)

Total RNA was extracted from ARPE‐19 cells using TRIzol reagent (Cat# 15596026, Invitrogen). RNA concentration and purity were assessed using the NanoDrop spectrophotometer (Thermo Fisher Scientific). A total of 1 μg RNA was utilized to synthesize cDNA with the PrimeScript RT reagent Kit with gDNA Eraser (Cat# RR047A, Takara Bio, Shiga, Japan). Quantitative PCR was performed using TB Green® Premix Ex Taq™ II (Cat# RR820A, Takara Bio) on a QuantStudio 5 real‐time PCR system (Applied Biosystems, Thermo Fisher Scientific). The expression levels of NR4A1, SMURF2, and FOXO6 were normalized to β‐actin, which served as an internal control. Relative gene expression was calculated using the 2^ΔΔCt^ method. Primer sequences for all genes are listed in Table 1.

**TABLE 1:** Sequences of the primers used in this study.

Primer Name	Sequence (5′→3′)	Sequence (5′→3′)
NR4A1	GGACAACGCTTCATGCCAGCAT	CCTTGTTAGCCAGGCAGATGTAC
SMURF2	TCCTCGGCTGTCTGCTAACTTG	CAGGCATTCTGTGTCATCAGGAC
FOXO6	AGACTCACGCTCTCGCAGATCT	GACAGGTTGTGCCGAATGGAGT
β‐actin	CACCATTGGCAATGAGCGGTTC	AGGTCTTTGCGGATGTCCACGT

### 2.4 Western blot analysis

Cells or retinal tissues were lysed in radioimmunoprecipitation assay (RIPA) buffer containing protease and phosphatase inhibitors (Cat# P0013B, Beyotime Biotechnology, Shanghai, China). Protein concentrations were assessed using a bicinchoninic acid (BCA) protein assay kit (Cat# P0010, Beyotime). Equal amounts of protein (20 μg) were separated by sodium dodecyl sulfate–polyacrylamide gel electrophoresis (SDS–PAGE) and transferred onto polyvinylidene difluoride (PVDF) membranes (Cat# IPVH00010, Millipore, Billerica, MA, USA). Membranes were blocked with 5% nonfat milk in TBST for 1 h at room temperature and incubated overnight at 4°C with the following primary antibodies: anti‐NR4A1 (1:1,000, Cat# ab153914, Abcam, Cambridge, UK), anti‐SMURF2 (1:1,000, Cat# ab313470, Abcam), anti‐FOXO6 (1:1,000, Cat# PA5‐35117, Thermo Fisher Scientific), anti‐GPX4 (1:1,000, Cat# MA5‐32827, Thermo Fisher Scientific), anti‐SLC7A11 (1:1,000, Cat# PA1‐16893, Thermo Fisher Scientific), anti‐ACSL4 (1:1,000, Cat# PA5‐27137, Thermo Fisher Scientific), anti‐OTUB1 (1:1,000, Cat# PA5‐58684, Thermo Fisher Scientific), anti‐OTUB2 (1:1,000, Cat# PA5‐99680, Thermo Fisher Scientific), and anti‐β‐actin (1:5,000, Cat# MA5‐42946, Thermo Fisher Scientific). After washing with TBST, membranes were incubated for 1 h at room temperature with HRP‐conjugated secondary antibodies (1:5,000, Cat# A‐11008, Thermo Fisher Scientific) and visualized using an enhanced chemiluminescence (ECL) detection kit (Cat# P0018FS, Beyotime). Signals were captured using a Tanon 5200 chemiluminescence imaging system (Tanon Science and Technology, Shanghai, China). Band intensities were quantified using ImageJ software (NIH, Bethesda, MD, USA) and normalized to β‐actin.

### 2.5 Cell viability assay

Cell viability was evaluated using the Cell Counting Kit‐8 (CCK‐8) assay (Cat# C0037, Beyotime Biotechnology). ARPE‐19 cells were seeded into 96‐well plates at a density of 5 × 10^3^ cells per well and cultured overnight. After experimental treatments, 10 μL of CCK‐8 solution was added to each well, followed by incubation at 37°C for 2 h. Absorbance was measured at 450 nm using a microplate reader (SpectraMax i3, Molecular Devices, San Jose, CA, USA). Cell viability was expressed as a percentage relative to the control group.

### 2.6 Measurement of ferrous iron (Fe^2+^), malondialdehyde (MDA), and superoxide dismutase (SOD) levels

Intracellular Fe^2+^, MDA, and SOD levels in ARPE‐19 cells and MDA and SOD levels in mouse retinal tissues were measured using commercial kits. Following treatment, cells were collected and lysed, and tissues were homogenized and centrifuged to obtain supernatants. Fe^2+^ content was determined using the Iron Assay Kit (Cat# ab83366, Abcam), MDA levels were measured using the MDA Assay Kit (Cat# A003‐1‐2, Nanjing Jiancheng Bioengineering Institute, Nanjing, China), and SOD activity was evaluated using the SOD Assay Kit (Cat# ab65354, Abcam), according to the manufacturer's protocols.

### 2.7 Measurement of intracellular Fe^2+^ using FerroOrange

Intracellular ferrous iron (Fe^2+^) accumulation was measured using the Fe^2+^‐selective fluorescent probe FerroOrange (Cat# F374; Dojindo, Kumamoto, Japan). ARPE‐19 cells were plated in 24‐well culture plates at a density of 1 × 10^5^ cells per well and subjected to the indicated experimental treatments. Cells were then rinsed twice with Hank's Balanced Salt Solution (HBSS) and incubated with 1 μM FerroOrange prepared in HBSS at 37°C for 30 min under light‐protected conditions. Following incubation, excess probe was removed by washing, and fluorescence signals were immediately captured using a fluorescence microscope with excitation and emission wavelengths set at 543 and 580 nm, respectively. Quantification of intracellular Fe^2+^ levels was performed by measuring relative fluorescence intensity using ImageJ software.

### 2.8 Assessment of lipid peroxidation using C11‐BODIPY 581/591 staining

Lipid peroxidation was measured using the fluorescent probe C11‐BODIPY 581/591 (Cat# D3861, Thermo Fisher Scientific). ARPE‐19 cells were seeded in 24‐well plates at a density of 1 × 10^5^ cells per well and subjected to the indicated treatments. After treatment, cells were washed twice with HBSS and incubated with 2 μM C11‐BODIPY 581/591 diluted in HBSS at 37°C for 30 min in the dark. Cells were then washed, harvested, and resuspended in HBSS for analysis. Fluorescence was analyzed using a BD FACSCanto™ II flow cytometer (BD Biosciences, San Jose, CA, USA), with excitation/emission settings of 488/510 nm for the oxidized form (green fluorescence) and 581/590 nm for the reduced form (red fluorescence). Lipid peroxidation levels were quantified as the percentage of oxidized C11‐BODIPY–positive cells or by calculating the ratio of green to red fluorescence intensity, using FlowJo software.

### 2.9 Co‐immunoprecipitation (Co‐IP) assays

Co‐IP was performed to assess NR4A1 ubiquitination and protein–protein interactions between NR4A1 and SMURF2, as well as NR4A1 and the deubiquitinating enzymes OTUB1/OTUB2 in ARPE‐19 cells. Cells were washed with cold PBS and lysed in IP lysis buffer (Cat# P0013, Beyotime) containing protease inhibitor cocktail (Cat# 04693159001, Roche, Basel, Switzerland) and 1 mM PMSF. Cell lysates were clarified by centrifugation at 12,000 g for 15 min at 4°C, and protein concentration was determined by BCA assay. For each IP, equal amounts of protein (500 μg) were pre‐cleared with Protein A/G magnetic beads (Cat# 88803, Thermo Fisher Scientific) for 1 h at 4°C with gentle rotation to reduce nonspecific binding. The pre‐cleared lysates were then incubated with the appropriate primary antibody, including anti‐NR4A1 (1:30, Cat# ab283264, Abcam), anti‐SMURF2 (1:50, Cat# MA5‐42771, Thermo Fisher Scientific), anti‐OTUB1 (1:20, Cat# RAB02039, Thermo Fisher Scientific), or anti‐OTUB2 (1:50, Cat# H00078990‐M14, Thermo Fisher Scientific), overnight at 4°C. A normal IgG control (1:100, Cat# MA5‐54746/MA5‐54746, Thermo Fisher Scientific) was included in parallel for each IP reaction to control for nonspecific binding. Following antibody binding, Protein A/G magnetic beads were introduced to the lysates and allowed to capture immune complexes by incubation at 4°C for 2 h. The beads were subsequently rinsed three to five times with ice‐cold lysis buffer to remove nonspecific interactions. Immunocomplexes were then released by heating the beads in 2× SDS sample buffer for 5 min. The eluted proteins were separated by SDS–PAGE and subjected to immunoblot analysis.

### 2.10 Glutathione S‐transferase (GST) pull‐down assay

A GST pull‐down assay was performed to examine the direct interaction between SMURF2 and NR4A1. Full‐length human SMURF2 cDNA was cloned into the pGEX‐4T‐1 vector (GE Healthcare, Chicago, IL, USA) to generate a GST‐SMURF2 fusion protein, which was transformed into *Escherichia coli* BL21 (DE3). Expression of the GST fusion protein was induced with 0.5 mM Isopropyl β‐D‐1‐thiogalactopyranoside (IPTG) for 4 h at 30°C. Bacterial cells were harvested and lysed by sonication in PBS containing a protease inhibitor cocktail (Cat# 04693159001, Roche, Basel, Switzerland). GST or GST‐SMURF2 was then purified by affinity chromatography using glutathione‐Sepharose 4B beads (Cat# 17‐0756‐01, GE Healthcare). ARPE‐19 cell lysates were prepared in NP‐40 lysis buffer (Cat# P0013F, Beyotime Biotechnology) supplemented with protease inhibitors. Equal amounts of ARPE‐19 lysate (500 μg total protein) were incubated with GST or GST‐SMURF2 beads at 4°C overnight with gentle rotation, allowing the “prey” (endogenous NR4A1) to bind the immobilized “bait.” After multiple washes with PBS to remove nonspecific binding, bound proteins were eluted by boiling in 2× SDS loading buffer. Eluates were separated by SDS‐PAGE and analyzed by Western blot using the anti‐NR4A1 antibody. GST alone served as a negative control to ensure the specificity of interaction.

### 2.11 Chromatin immunoprecipitation (ChIP) assay

A ChIP assay was conducted to assess the binding of FOXO6 to the SMURF2 promoter using a commercial ChIP kit (Cat# 17–295, Millipore) following the manufacturer's instructions. ARPE‐19 cells were seeded in 10 cm dishes at a density of 1 × 10^6^ cells per dish and subjected to the indicated treatments. Cells were cross‐linked with 1% formaldehyde at room temperature for 10 min to fix DNA–protein interactions, followed by quenching with 125 mM glycine for 10 min. Nuclei were extracted, and chromatin was sheared to 200–1000 bp fragments by sonication (10 cycles of 30 s on/30 s off, on ice). The fragmented chromatin was immunoprecipitated overnight at 4°C using a FOXO6 antibody (1:50, Cat# PA5‐35117, Invitrogen) or negative control rabbit IgG (1:100, Cat# MA5‐56524, Thermo Fisher Scientific) antibody along with protein A/G magnetic beads. After reverse crosslinking at 65°C, DNA was purified using spin columns. The enrichment of FOXO6 binding at the SMURF2 promoter region was analyzed by qPCR. Results were expressed as a percentage of input DNA and normalized to the IgG control.

### 2.12 Dual‐luciferase reporter assay

The effect of FOXO6 knockdown on SMURF2 promoter activity was assessed using a dual‐luciferase reporter assay. The human SMURF2 promoter region containing predicted FOXO6 binding sites was amplified by PCR and cloned into the pGL3‐basic luciferase reporter vector (Cat# E1751, Promega, Madison, WI, USA) to generate pGL3‐SMURF2‐WT. A mutant construct (pGL3‐SMURF2‐Mut) was generated by site‐directed mutagenesis of the FOXO6 binding motif using a Mut Express II Fast Mutagenesis Kit V2 (Cat# C214‐01, Vazyme, Nanjing, China). ARPE‐19 cells were then seeded into 24‐well plates at a density of 5 × 10^4^ cells/well and co‐transfected with 0.8 μg of pGL3‐WT or pGL3‐Mut, 0.02 μg of pRL‐TK Renilla luciferase plasmid (Cat# E2241, Promega) as an internal control, and either sh‐NC or sh‐FOXO6 plasmids using Lipofectamine 3000. After 48 h, luciferase activity was measured using the Dual‐Luciferase Reporter Assay System (Cat# E1910, Promega) on a GloMax 96 Microplate Luminometer (Promega). Firefly luciferase activity was normalized to Renilla luciferase activity. Data were presented as relative luciferase activity.

### 2.13 DM mouse model establishment and body weight monitoring

Male C57BL/6 mice (6–8 weeks old, 18–22 g) were purchased from Hunan Sleke Jingda Laboratory Animal Co., LTD (Hunan, China). All animal procedures complied with the Guide for the Care and Use of Laboratory Animals and were approved by the Ethics Committee of Health Science Center, Yangtze University. Mice were randomly assigned to control and diabetic groups. The DM model was induced based on a previously published protocol with modifications.^9^ Mice in the diabetic group were fed a high‐fat diet (HFD; 60% kcal from fat) for 8 weeks. After an overnight fast (10 h), an intraperitoneal glucose tolerance test (IPGTT) was performed: 20% glucose (2 g/kg) was injected i.p. and blood glucose was measured from the tail vein at 0, 15, 30, 60, 90, and 120 min. Following HFD and glucose testing, mice received streptozotocin (STZ; 55 mg/kg/day) intraperitoneally for 5 consecutive days (STZ freshly prepared in 0.1 M citrate buffer, pH 4.5). Control mice received citrate buffer alone. Fasting blood glucose was measured from the tail vein 3 days after the final STZ injection; mice with stable glucose >11.1 mmol/L for at least 5 days were considered diabetic. Lentiviral vectors carrying oe‐SMURF2, oe‐NR4A1, oe‐NC, sh‐FOXO6, sh‐SMURF2, or sh‐NC were constructed and packaged by GeneChem Co., Ltd. (Shanghai, China) using a third‐generation packaging system, as previously described.^20^ Viral titers were adjusted to 1 × 10^9^ TU/mL. For in vivo gene delivery, intravitreal injections were performed under anesthesia (1% pentobarbital sodium) using a microsyringe with a 33‐gauge needle inserted into the vitreous cavity; 2 μL lentiviral suspension was injected per eye. Injections were administered on Day 1, Week 1, and Week 2 post‐STZ to achieve stable retinal transduction. Body weight was measured weekly using an electronic balance at Weeks 4, 8, and 12.

### 2.14 Blood glucose level measurement

Fasting blood glucose levels were assessed using a glucometer (Yuwell 580, Yuwell, Jiangsu, China) from the tail vein blood after a 10‐h overnight fast. Mice with fasting blood glucose levels exceeding 11.1 mmol/L for at least five consecutive days were considered diabetic. For dynamic glucose monitoring at Weeks 4, 8, and 12, additional blood glucose measurements were performed using the same method.

### 2.15 Hematoxylin and eosin staining

Paraffin‐embedded mouse eyeballs were sectioned at a thickness of 5 μm along the sagittal plane through the optic nerve. Sections were deparaffinized, rehydrated, and stained with H&E following standard protocols to assess retinal morphology. Images were captured using a light microscope (Nikon Eclipse Ci, Nikon, Tokyo, Japan). To evaluate RPE pathology, the integrity of retinal layers and RPE structure was assessed. The number of RPE cells was quantified in a defined region (approximately 250 μm adjacent to the optic nerve) in three nonoverlapping fields per section. Cell counts were performed in a blinded manner using the ImageJ software.

### 2.16 Immunofluorescence staining

Paraffin‐embedded mouse eyeball sections were used for immunofluorescence staining to examine the expression of SMURF2 and NR4A1 in the RPE layer. Briefly, retinal sections were deparaffinized in xylene, rehydrated through a graded ethanol series, and subjected to antigen retrieval using citrate antigen retrieval buffer (Cat# P0081, Beyotime Biotechnology). After washing with PBS, the sections were permeabilized with 0.3% Triton X‐100 (Cat# T8787, Sigma‐Aldrich) for 15 min and blocked with 5% bovine serum albumin (BSA; Cat# A7906, Sigma‐Aldrich) for 1 h at room temperature. The sections were then incubated overnight at 4°C with anti‐SMURF2 antibody (1:500, Cat# MA5‐42771, Invitrogen) and anti‐NR4A1 antibody (1:200, Cat# 14‐5965‐82, Invitrogen). After three washes with PBS, the sections were incubated with Alexa Fluor 488‐conjugated goat anti‐mouse IgG secondary antibody (1:500, Cat# A‐11001, Thermo Fisher Scientific) and Alexa Fluor 568‐conjugated goat anti‐rabbit IgG secondary antibody (1:500, Cat# A‐11011, Thermo Fisher Scientific) for 1 h at room temperature in the dark. Nuclei were counterstained with DAPI (Cat# D1306, Thermo Fisher Scientific). Finally, the sections were mounted with anti‐fade mounting medium (Cat# P36930, Thermo Fisher Scientific), and images were captured using a fluorescence microscope under identical acquisition settings. The colocalization of SMURF2 and NR4A1 in the RPE layer was assessed based on merged fluorescence signals.

### 2.17 Bioinformatics analyses

Bioinformatics analyses were performed to inform experimental design and identify candidate interactions. The BioGRID database (https://thebiogrid.org/) was queried to identify proteins that have been reported to interact with NR4A1, with particular attention to enzymes involved in ubiquitination and deubiquitination; the resulting candidate list guided selection of SMURF2, OTUB1, and OTUB2 for empirical testing. To predict potential transcriptional regulation of SMURF2 by FOXO6, the JASPAR database (https://jaspar.genereg.net/) was used to scan the SMURF2 promoter region for putative FOXO6 binding motifs using the CORE vertebrate profile collection.

### 2.18 Statistical analysis

All quantitative data are presented as the mean ± standard deviation from at least three independent experiments. Statistical analysis was performed using GraphPad Prism 9.0 (GraphPad Software, San Diego, CA, USA). For comparisons between two groups, the unpaired two‐tailed Student's *t*‐test was used. For comparisons among multiple groups, one‐way or two‐way analysis of variance followed by Tukey's post hoc test was applied. A *p*‐value <0.05 was considered statistically significant.

## 3 RESULTS

### 3.1 NR4A1 knockdown attenuated high glucose‐induced ferroptosis in retinal pigment epithelial cells

**FIGURE 1:**
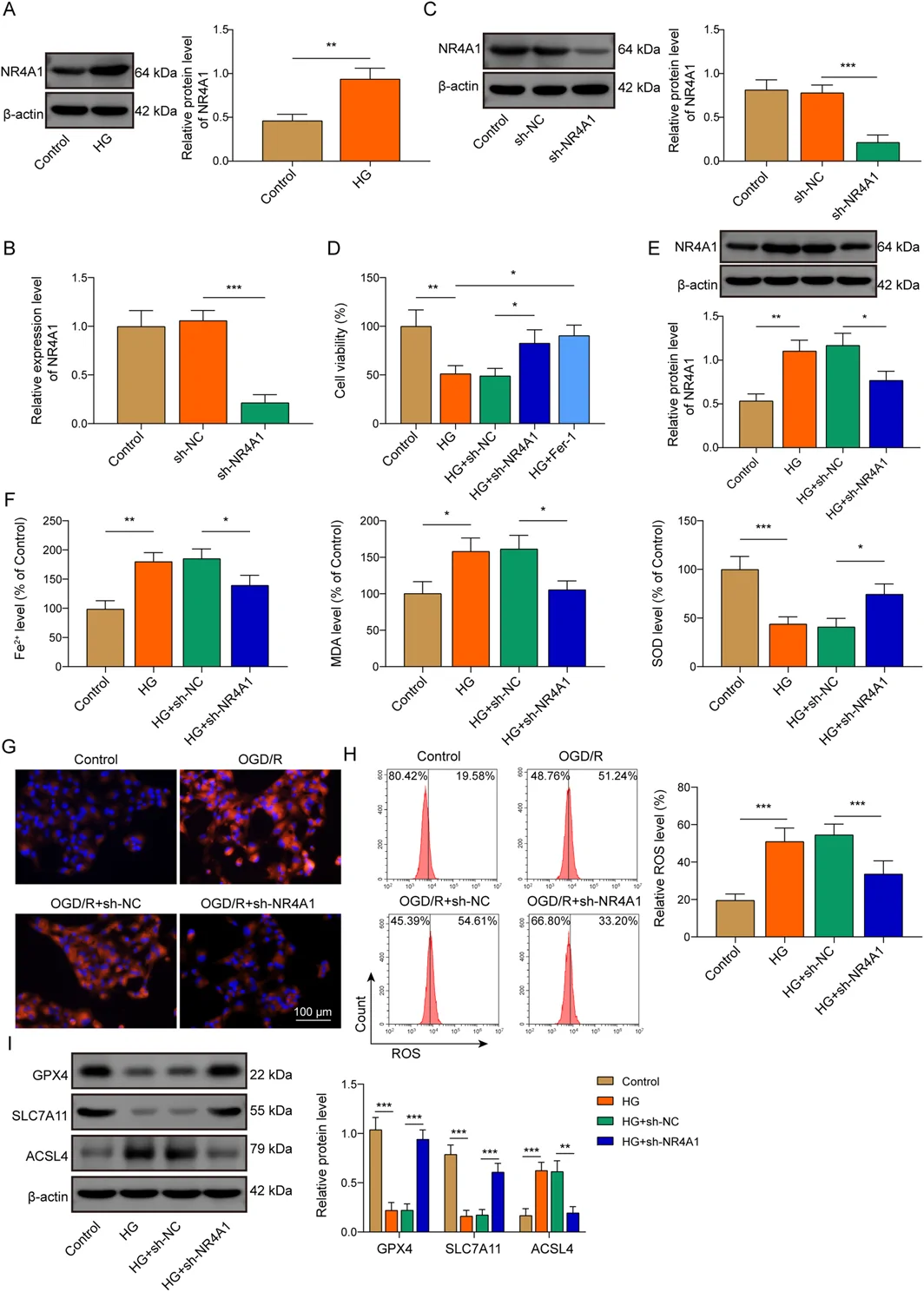
NR4A1 knockdown attenuated high glucose‐induced ferroptosis in retinal pigment epithelial cells. (A) Western blot analysis of NR4A1 expression in ARPE‐19 cells under control and HG conditions. (B, C) Validation of NR4A1 knockdown in ARPE‐19 cells using RT‐qPCR (B) and Western blot (C) following transfection with sh‐NR4A1 or sh‐NC. (D) Cell viability was assessed by CCK‐8 assay in cells treated with HG, sh‐NR4A1, or the ferroptosis inhibitor Fer‐1. (E–I) ARPE‐19 cells were subjected to NR4A1 knockdown followed by HG induction. (E) Western blot analysis of NR4A1 expression. (F) Measurement of intracellular Fe^2+^, MDA, and SOD using commercial assay kits. (G) Detection of intracellular Fe^2+^ using the FerroOrange fluorescent probe. (H) Assessment of lipid peroxidation levels using C11‐BODIPY 581/591 staining. (I) Western blot analysis of ferroptosis‐related proteins GPX4, SLC7A11, and ACSL4. *n* = 3. Data are presented as mean ± SD. *p < 0.05, **p < 0.01, ***p < 0.001. HG, High glucose.

To investigate the role of NR4A1 in HG‐induced ferroptosis in RPE cells, we first assessed NR4A1 expression in ARPE‐19 cells. NR4A1 protein was significantly elevated in response to HG treatment (Figure 1A). To explore its functional relevance, NR4A1 was silenced using shRNA, and efficient knockdown was validated at the mRNA and protein levels (Figure 1B,C). HG significantly reduced cell viability, whereas NR4A1 knockdown or treatment with the ferroptosis inhibitor Fer‐1 restored viability (Figure 1D). Furthermore, NR4A1 knockdown reversed HG‐induced increases in NR4A1 expression, intracellular Fe^2+^ levels, and the lipid peroxidation marker MDA, and restored antioxidant enzyme SOD activity (Figure 1E,F). Consistently, NR4A1 silencing reduced HG‐induced Fe^2+^ accumulation (Figure 1G). Lipid peroxidation levels, elevated by HG, were significantly decreased following NR4A1 knockdown (Figure 1H). Lastly, HG downregulated ferroptosis‐inhibiting proteins GPX4 and SLC7A11, and upregulated the ferroptosis‐promoting enzyme ACSL4 (Figure 1I). These alterations were alleviated by NR4A1 knockdown. Collectively, NR4A1 contributed to HG‐induced ferroptosis in RPE cells, and its inhibition protected against ferroptosis.

### 3.2 SMURF2 facilitated the ubiquitin‐mediated degradation of NR4A1 in retinal pigment epithelial cells

**FIGURE 2:**
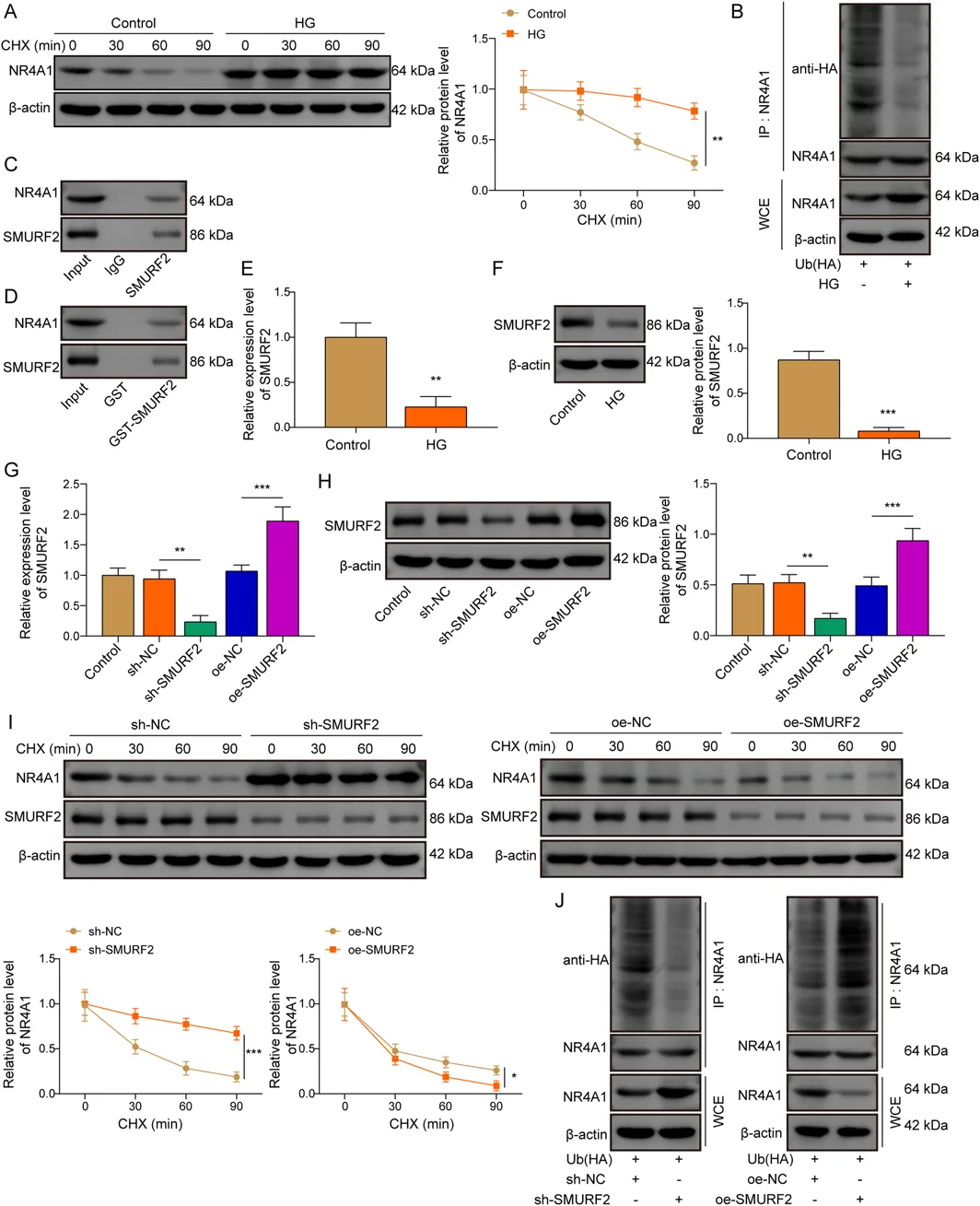
SMURF2 facilitated the ubiquitin‐mediated degradation of NR4A1 in retinal pigment epithelial cells. (A) CHX chase assay to assess NR4A1 protein stability in ARPE‐19 cells under control and HG conditions. (B) Co‐IP analysis of NR4A1 ubiquitination levels in ARPE‐19 cells treated with or without HG. (C, D) Co‐IP (C) and GST pull‐down (D) assays detected the interaction between SMURF2 and NR4A1 in ARPE‐19 cells. (E, F) RT‐qPCR (E) and Western blot (F) analysis of SMURF2 expression in control and HG‐treated ARPE‐19 cells. (G–J) ARPE‐19 cells were subjected to SMURF2 knockdown or overexpression by transfection with sh‐SMURF2 or oe‐SMURF2, respectively, with corresponding negative controls (sh‐NC or oe‐NC). RT‐qPCR (G) and Western blot (H) validation of SMURF2 knockdown and overexpression efficiency. (I) CHX chase assays evaluating NR4A1 protein stability in ARPE‐19 cells with SMURF2 knockdown or overexpression. (J) Co‐IP analysis of NR4A1 ubiquitination levels following SMURF2 knockdown or overexpression in ARPE‐19 cells. *n* = 3. Data are presented as mean ± SD. *p < 0.05, **p < 0.01, ***p < 0.001. GST, Glutathione S‐transferase; HG, High glucose.

CHX chase assay found that NR4A1 exhibited increased stability in the HG group compared to the control group, indicating a slower degradation rate (Figure 2A). HG treatment markedly reduced the ubiquitination of NR4A1 (Figure 2B), suggesting that the ubiquitin‐mediated degradation of NR4A1 is inhibited under HG conditions. To identify potential ubiquitin regulators, we used the BioGRID database to predict the proteins that interact with NR4A1 (Supporting Information S1: Figure S1A). Through this screening, we focused on the ubiquitination‐related enzymes among them, specifically the E3 ubiquitin ligase SMURF2 and the deubiquitinating enzymes OTUB1 and OTUB2 (Supporting Information S1: Figure S1B). To identify which protein has an interaction with NR4A1, we used Co‐IP and GST pull‐down assays, both of which confirmed a interaction between SMURF2 and NR4A1 (Figure 2C,D). In contrast, Co‐IP analysis showed no detectable interaction between NR4A1 and OTUB1 or OTUB2 (Supporting Information S1: Figure S1C,D), suggesting these deubiquitinating enzymes do not regulate NR4A1 in RPE cells. Furthermore, SMURF2 mRNA and protein expression was significantly downregulated in ARPE‐19 cells under HG conditions (Figure 2E,F). To evaluate the regulatory effect of SMURF2 on NR4A1, SMURF2 was knocked down or overexpressed in ARPE‐19 cells. As seen in Figure 2G,H, efficient knockdown and overexpression were confirmed. SMURF2 knockdown prolonged NR4A1 protein half‐life, whereas SMURF2 overexpression accelerated its degradation (Figure 2I). Consistently, Co‐IP experiments showed that SMURF2 knockdown reduced NR4A1 ubiquitination, whereas SMURF2 overexpression enhanced it (Figure 2J). These findings indicate that SMURF2 interacted with NR4A1 and promoted its ubiquitination and degradation in RPE cells.

### 3.3 Overexpression of NR4A1 reversed the protective effect of SMURF2 overexpression against high glucose‐induced ferroptosis in retinal pigment epithelial cells

**FIGURE 3:**
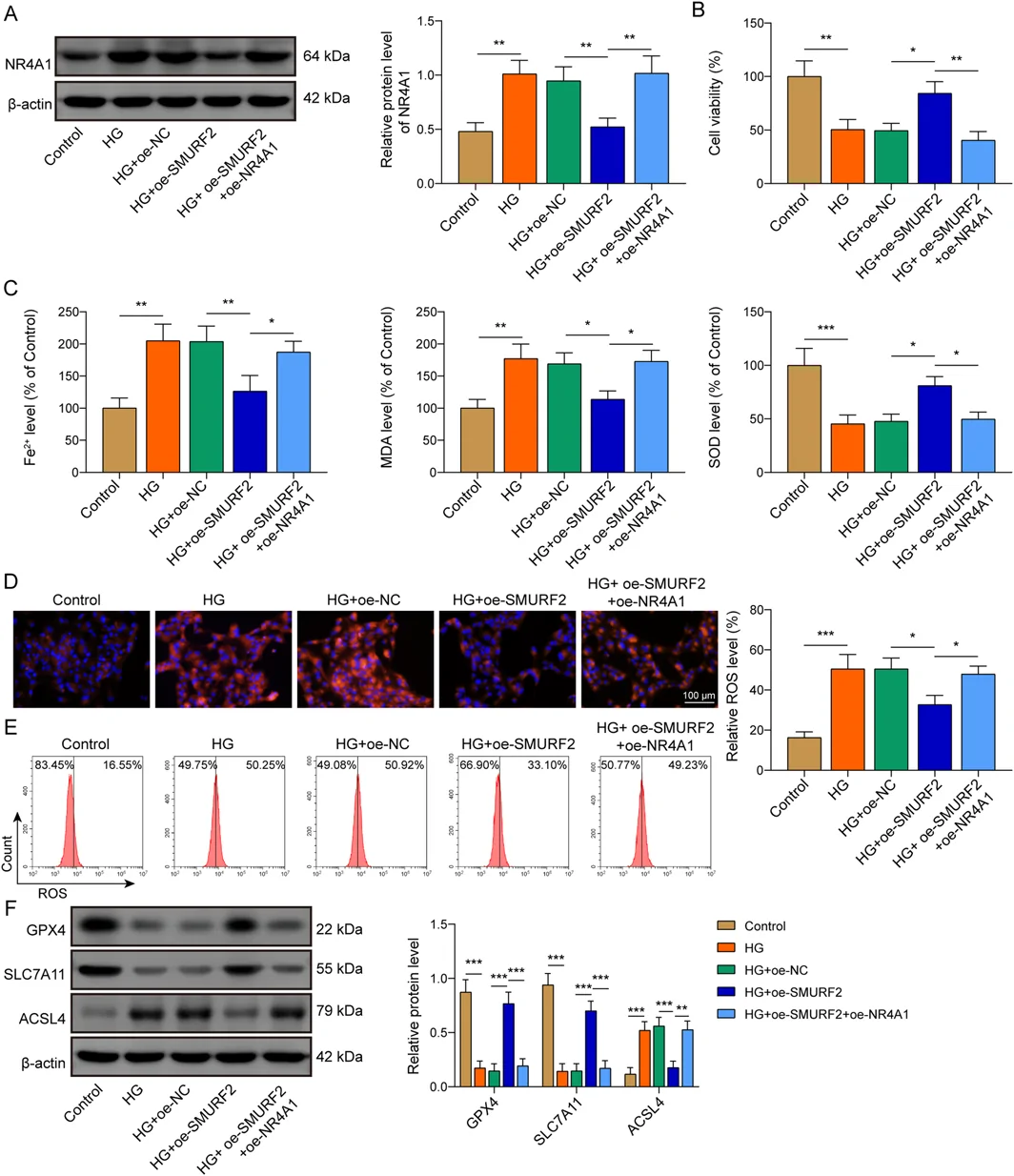
Overexpression of NR4A1 reversed the protective effect of SMURF2 overexpression against high glucose‐induced ferroptosis in retinal pigment epithelial cells. (A–F) ARPE‐19 cells were first transfected with oe‐SMURF2 alone or in combination with oe‐NR4A1, and subsequently exposed to HG conditions. Untreated cells maintained under NG served as controls. (A) Western blot analysis of NR4A1 expression in ARPE‐19 cells. (B) Cell viability was assessed by the CCK‐8 assay. (C) Measurement of intracellular Fe^2+^, MDA, and SOD levels using commercial assay kits. (D) Detection of intracellular Fe^2+^ using the FerroOrange fluorescent probe. (E) Assessment of lipid peroxidation levels using C11‐BODIPY 581/591 fluorescence staining. (F) Western blot analysis of ferroptosis‐related proteins GPX4, SLC7A11, and ACSL4. *n* = 3. Data are presented as mean ± SD. *p < 0.05, **p < 0.01, ***p < 0.001. HG, High glucose.

To determine whether NR4A1 mediates the effects of SMURF2 on ferroptosis, we examined the impact of SMURF2 overexpression in ARPE‐19 cells under HG conditions with or without NR4A1 overexpression. SMURF2 overexpression reduced HG‐induced NR4A1 protein levels, and this reduction was restored by co‐overexpression of NR4A1 (Figure 3A). SMURF2 overexpression increased cell viability in HG‐treated cells, whereas co‐overexpression of NR4A1 suppressed this effect (Figure 3B). Similarly, SMURF2 overexpression reversed the HG‐induced elevation of intracellular Fe^2+^ and MDA levels and the reduction in SOD activity, and these effects were abolished when NR4A1 was co‐overexpressed (Figure 3C). SMURF2 overexpression reduced the HG‐induced increase in Fe^2+^ accumulation, while NR4A1 co‐overexpression restored Fe^2+^ levels (Figure 3D). In addition, SMURF2 overexpression lowered HG‐induced lipid peroxidation accumulation, whereas NR4A1 co‐overexpression reversed this effect (Figure 3E). SMURF2 overexpression counteracted HG‐induced changes in ferroptosis‐related proteins GPX4, SLC7A11, and ACSL4, and that NR4A1 co‐overexpression negated these regulatory effects (Figure 3F). Together, NR4A1 functionally opposed the SMURF2‐mediated suppression of HG‐induced ferroptosis in RPE cells.

### 3.4 NR4A1 overexpression attenuated the effect of SMURF2 overexpression on ferroptosis in the retina of diabetic mice

**FIGURE 4:**
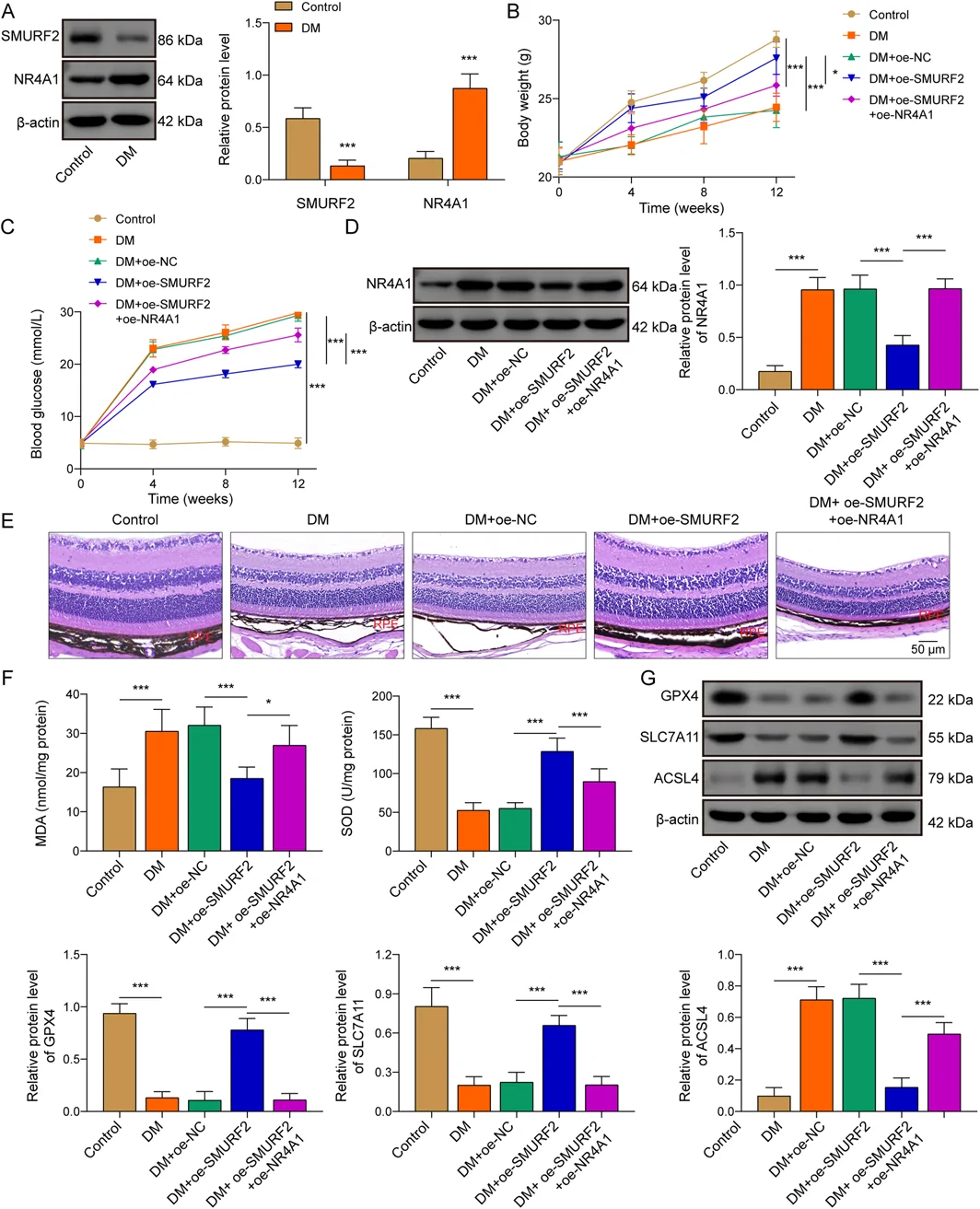
NR4A1 overexpression attenuated the effect of SMURF2 overexpression on ferroptosis in the retina of diabetic mice. (A) Western blot analysis of SMURF2 and NR4A1 protein levels in retinal tissues of control and DM mice. (B–G) DM mice were subjected to oe‐SMURF2 alone or in combination with oe‐NR4A1 using the corresponding control vectors, whereas nondiabetic mice served as controls. (B, C) Body weight (B) and blood glucose levels (C) were monitored in different groups of mice over 12 weeks. (D) Western blot analysis of NR4A1 protein levels in retinal tissues across treatment groups. (E) Histopathological changes in the retina were assessed by H&E staining. Representative images show structural alterations and RPE cell loss. Scale bar = 50 μm. (F) MDA and SOD levels in retinal tissues were measured using commercial assay kits. (G) Western blot analysis of ferroptosis‐related proteins GPX4, SLC7A11, and ACSL4 in retinal tissues. *n* = 6. Data are presented as mean ± SD. *p < 0.05, ***p < 0.001.

Based on the in vitro findings, we next investigated whether the SMURF2–NR4A1 regulatory axis modulates ferroptosis in vivo using a DM mouse model. Western blot analysis of retinal tissues from DM mice showed that SMURF2 protein levels were reduced, whereas NR4A1 levels were elevated compared to controls (Figure 4A). To assess the functional role of this regulatory axis in vivo, SMURF2 and/or NR4A1 were overexpressed in DM mice using intravitreal injection of lentiviral vectors. SMURF2 overexpression partially promoted body weight and reduced blood glucose levels in DM mice, whereas co‐overexpression of NR4A1 abolished these effects (Figure 4B,C). NR4A1 protein levels, which were decreased by SMURF2 overexpression, were restored by co‐overexpression of NR4A1 (Figure 4D). Immunofluorescence staining further showed that SMURF2 expression was reduced in the RPE layer of DM mice, whereas NR4A1 expression was increased, with relatively weak colocalization between the two proteins. SMURF2 overexpression restored SMURF2 expression and enhanced SMURF2–NR4A1 colocalization while reducing NR4A1 levels; however, NR4A1 co‐overexpression increased NR4A1 expression without further elevating SMURF2 expression (Supporting Information S1: Figure S2). Histological analysis revealed that SMURF2 overexpression preserved retinal structure and RPE integrity, whereas NR4A1 co‐overexpression reversed these protective effects, manifested by retinal thinning, loss of RPE cells, and disrupted tissue organization (Figure 4E). Similarly, SMURF2 overexpression lowered MDA levels and increased SOD activity in the retina, whereas NR4A1 co‐overexpression reversed these changes (Figure 4F). The restoration of GPX4 and SLC7A11 levels and suppression of ACSL4 by SMURF2 overexpression were negated by NR4A1 co‐overexpression (Figure 4G). Altogether, NR4A1 mediated the pro‐ferroptotic changes observed in DR and counteracted the protective effects of SMURF2 in vivo.

### 3.5 FOXO6 transcriptionally repressed SMURF2 expression in retinal pigment epithelial cells

**FIGURE 5:**
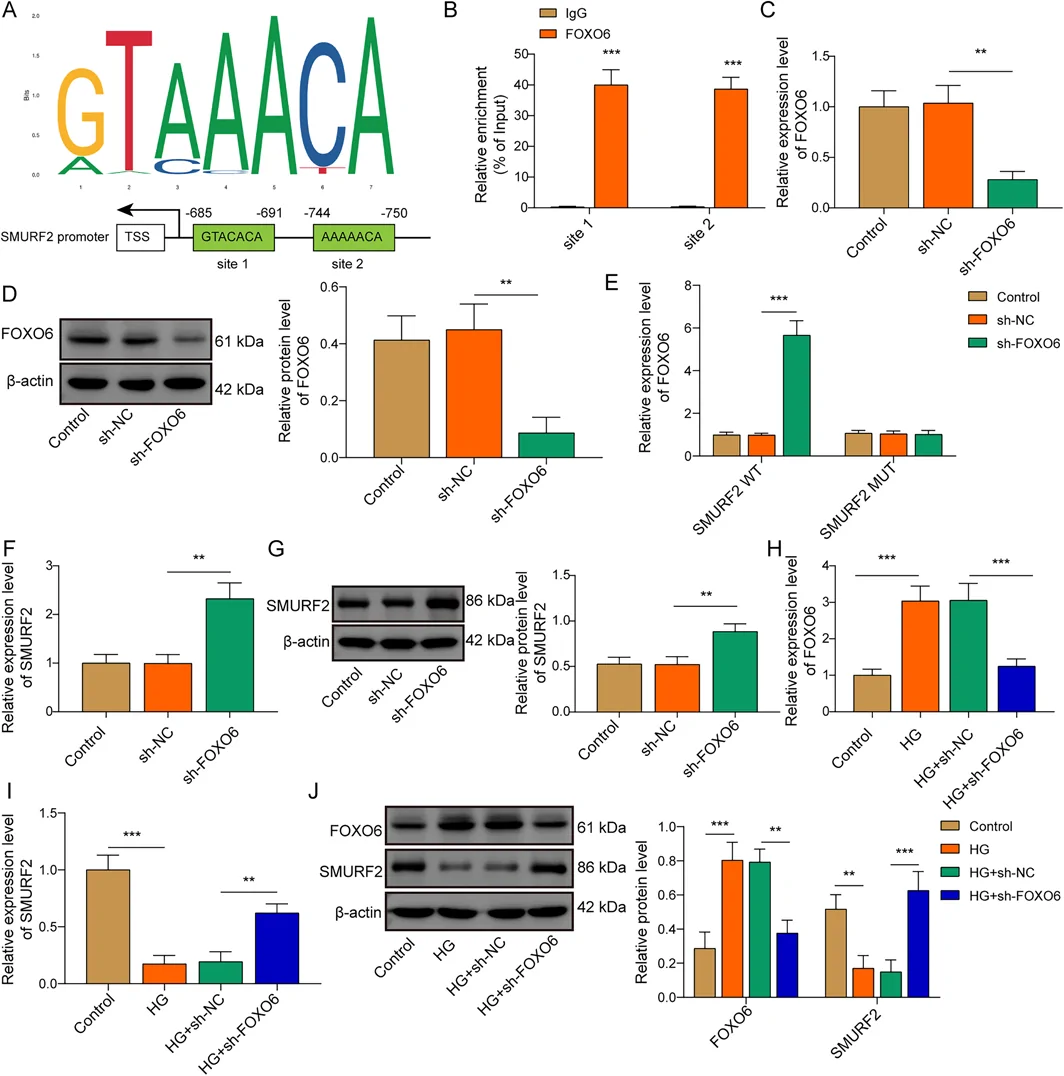
FOXO6 transcriptionally repressed SMURF2 expression in retinal pigment epithelial cells. (A) Predicted FOXO6 binding motifs in the SMURF2 promoter region identified using the JASPAR database. (B) ChIP assay detected the binding of FOXO6 to the SMURF2 promoter in ARPE‐19 cells. (C, D) RT‐qPCR (C) and Western blot (D) analysis of FOXO6 expression following shRNA‐mediated knockdown in ARPE‐19 cells. (E) Dual‐Luciferase Reporter Assay examined SMURF2 promoter activity using wild‐type and mutant promoter constructs after FOXO6 knockdown. (F, G) RT‐qPCR (F) and Western blot (G) analysis of SMURF2 expression after FOXO6 knockdown in ARPE‐19 cells. (H–J) Cells were first transfected with sh‐FOXO6 or sh‐NC and subsequently exposed to HG conditions, whereas cells maintained under NG served as controls. (H, I) RT‐qPCR analysis of FOXO6 (H) and SMURF2 (I) expression in ARPE‐19 cells. (J) Western blot analysis of FOXO6 and SMURF2 protein levels. *n* = 3. Data are presented as mean ± SD. **p < 0.01, ***p < 0.001. HG, High glucose.

FOXO6 has been reported to be upregulated in vitreous samples from patients with DR and in HG‐treated ARPE‐19 cells, where its knockdown attenuates oxidative stress and apoptosis via Akt/Nrf2 signaling.^19^ To explore the transcriptional regulation of SMURF2, JASPAR database analysis predicted two potential FOXO6 binding sites within the SMURF2 promoter region (Figure 5A). ChIP assays confirmed significant enrichment of FOXO6 binding at both predicted sites, indicating a direct interaction between FOXO6 and the SMURF2 promoter (Figure 5B). To evaluate the functional consequences of this interaction, FOXO6 was knocked down in ARPE‐19 cells using shRNA. FOXO6 expression was significantly decreased upon transfection with sh‐FOXO6 (Figure 5C,D). Dual‐luciferase reporter assays showed that FOXO6 knockdown significantly increased SMURF2 promoter activity, whereas mutation of the FOXO6 binding sites abolished this effect (Figure 5E). Consistent with the reporter assay, FOXO6 knockdown led to upregulation of SMURF2 expression (Figure 5F,G). Under HG conditions, FOXO6 expression was elevated, and SMURF2 expression was suppressed. Importantly, FOXO6 knockdown reversed the HG‐induced upregulation of FOXO6 and downregulation of SMURF2 (Figure 5H–J). Considering that SMURF2 functions as an E3 ubiquitin ligase, we further examined whether SMURF2 might reciprocally regulate FOXO6 through direct protein interaction. Co‐IP analysis in ARPE‐19 cells did not detect an interaction between SMURF2 and FOXO6 proteins, arguing against the possibility that SMURF2 directly regulates FOXO6 protein stability through binding‐dependent ubiquitination (Supporting Information S1: Figure S3). These findings demonstrate that FOXO6 directly bound to the SMURF2 promoter and negatively regulated its transcription in RPE cells.

### 3.6 SMURF2 knockdown reversed the protective effect of FOXO6 knockdown against high glucose‐induced ferroptosis in retinal pigment epithelial cells

**FIGURE 6:**
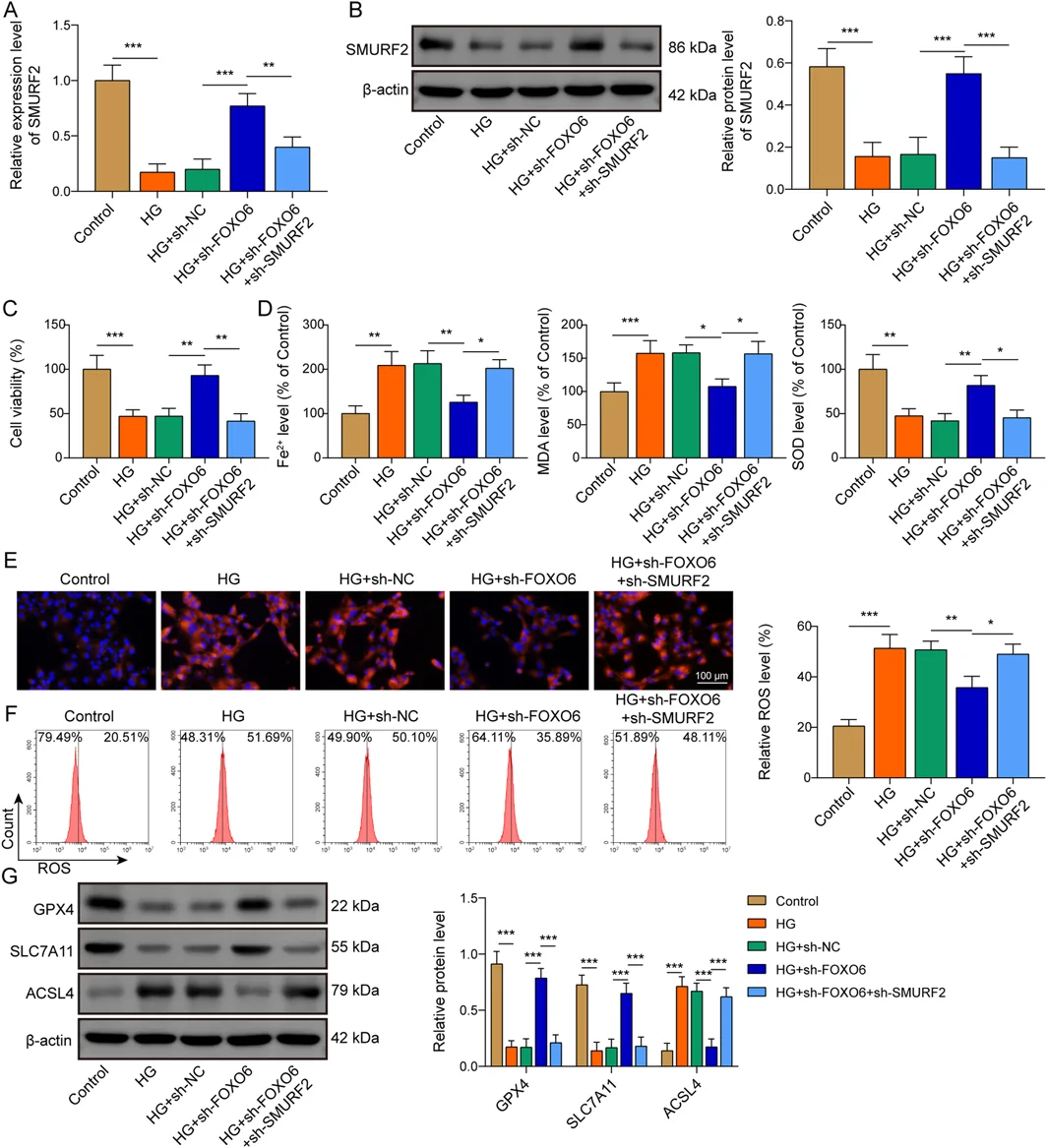
SMURF2 knockdown reversed the protective effect of FOXO6 knockdown against high glucose‐induced ferroptosis in retinal pigment epithelial cells. (A–G) ARPE‐19 cells were first transfected with sh‐FOXO6 alone or in combination with sh‐SMURF2, or sh‐NC, and subsequently exposed to HG conditions. Cells maintained under NG served as controls. RT‐qPCR (A) and Western blot (B) analysis of SMURF2 expression in ARPE‐19 cells. (C) Cell viability was assessed using the CCK‐8 assay. (D) Quantification of intracellular Fe^2+^, malondialdehyde (MDA), and superoxide dismutase (SOD) levels using commercial assay kits. (E) Detection of intracellular Fe^2+^ using the FerroOrange fluorescent probe. (F) Analysis of lipid peroxidation levels using C11‐BODIPY 581/591 fluorescence staining. (G) Western blot analysis of ferroptosis‐related proteins GPX4, SLC7A11, and ACSL4 under the indicated treatment condition. *n* = 3. Data are presented as mean ± SD. *p < 0.05, **p < 0.01, ***p < 0.001. HG, High glucose.

To investigate whether SMURF2 mediates the regulatory effects of FOXO6 on ferroptosis, we performed co‐knockdown experiments in ARPE‐19 cells under HG conditions. FOXO6 knockdown restored SMURF2 expression suppressed by HG, whereas co‐knockdown of SMURF2 effectively reduced SMURF2 levels (Figure 6A,B). The enhancement of cell viability by FOXO6 knockdown was significantly reversed when SMURF2 was co‐silenced (Figure 6C). FOXO6 knockdown reduced Fe^2+^ and MDA levels and restored SOD activity, whereas SMURF2 co‐knockdown abolished these effects (Figure 6D). Consistently, FerroOrange fluorescent probe assay found that the reduction in Fe^2+^ levels induced by FOXO6 knockdown was reversed upon SMURF2 silencing (Figure 6E). Lipid peroxidation levels, suppressed by FOXO6 knockdown, were re‐elevated following SMURF2 knockdown (Figure 6F). FOXO6 knockdown restored GPX4 and SLC7A11 levels and reduced ACSL4 expression, whereas co‐knockdown of SMURF2 reversed these regulatory effects (Figure 6G). Collectively, SMURF2 acted downstream of FOXO6 to modulate ferroptosis in RPE cells under HG conditions.

### 3.7 SMURF2 knockdown reversed the inhibitory effect of FOXO6 knockdown against ferroptosis in diabetic mouse retinas

**FIGURE 7:**
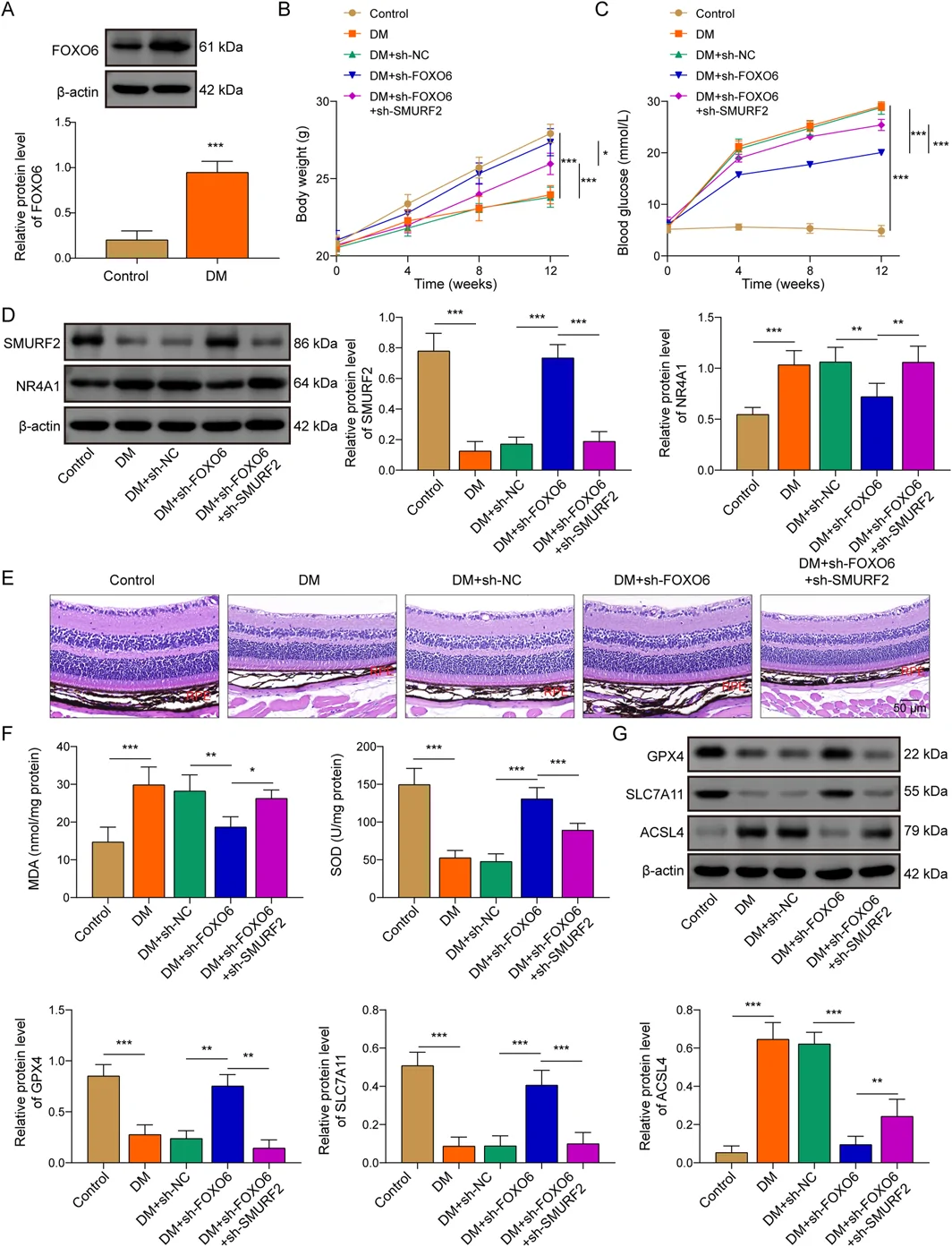
SMURF2 knockdown reversed the inhibitory effect of FOXO6 knockdown against ferroptosis in diabetic mouse retinas. (A) Western blot analysis of FOXO6 protein expression in retinal tissues from control and DM mice. (B–H) Male C57BL/6 mice were subjected to diabetes induction and then received sh‐FOXO6 vectors alone or in combination with sh‐SMURF2 or sh‐NC. Nondiabetic mice served as controls. (B, C) Body weight (B) and blood glucose levels (C) were monitored over a 12‐week period. (D) Western blot analysis of SMURF2 and NR4A1 protein levels in retinal tissues. (E) Retinal histopathological alterations were assessed by H&E staining. Representative images show changes in the retinal structure and RPE integrity. Scale bar = 50 μm. (F) MDA and SOD levels in retinal tissues were measured using commercial assay kits. (G) Western blot analysis of ferroptosis‐related proteins, including GPX4, SLC7A11, and ACSL4, in retinal tissues. *n* = 6. Data are presented as mean ± SD. *p < 0.05, **p < 0.01, ***p < 0.001.

To examine whether SMURF2 mediates the effects of FOXO6 on ferroptosis in vivo, we performed co‐knockdown experiments in a DM mouse model. FOXO6 protein levels were elevated in the retinas of DM mice compared to controls (Figure 7A). FOXO6 knockdown led to an increase in body weight and a decrease in blood glucose levels in DM mice, while additional SMURF2 knockdown partially reversed these effects (Figure 7B,C). SMURF2 expression was reduced in DM retinas and restored by FOXO6 knockdown, whereas co‐knockdown of SMURF2 suppressed SMURF2 expression; NR4A1 levels, which were increased in DM retinas, were downregulated by FOXO6 knockdown and restored by SMURF2 co‐knockdown (Figure 7D). FOXO6 knockdown improved the retinal structure and increased the number of RPE cells, whereas SMURF2 knockdown reversed these protective changes (Figure 7E). What's more, FOXO6 knockdown decreased MDA levels and increased SOD activity in retinal tissue, whereas SMURF2 knockdown abolished these effects (Figure 7F). Finally, FOXO6 knockdown restored GPX4 and SLC7A11 expression and suppressed ACSL4, whereas co‐knockdown of SMURF2 reversed these regulatory changes (Figure 7G). Taken together, the protective effects of FOXO6 depletion against ferroptosis in DM mice were abolished upon SMURF2 knockdown.

## 4 DISCUSSION

DR remains a main cause of blindness in working‐age populations.^2^ Despite the development of anti‐VEGF agents and improved glycemic control strategies, many patients still experience progressive retinal damage.^21^ This highlights the need for a deeper understanding of the cellular and molecular mechanisms driving DR pathogenesis. In this study, we uncovered a novel regulatory axis, FOXO6/SMURF2/NR4A1, that modulates ferroptosis in RPE cells and contributes to DR progression. Our data demonstrate that FOXO6 suppressed SMURF2 transcription, thereby stabilizing NR4A1 by reducing its ubiquitin‐mediated degradation. Accumulated NR4A1 then promoted ferroptosis, exacerbating RPE injury under hyperglycemic conditions. These findings provide mechanistic insights into ferroptosis regulation in DR and identify SMURF2 as a potential therapeutic target.

Under diabetic conditions, hyperglycemia induces a range of pathological alterations in RPE cells, including oxidative stress, mitochondrial dysfunction, epithelial barrier disruption, and inflammatory activation.^22^ These changes compromise the physiological functions of RPE cells and disrupt retinal homeostasis, contributing to damage of the neurovascular unit—a hallmark of DR pathogenesis.^23^ Emerging evidence has identified ferroptosis as a key contributor to RPE degeneration in diabetes.^7^ Both RPE and endothelial cells have been shown to undergo ferroptosis under hyperglycemic conditions, promoting enhanced vascular permeability and breakdown of the BRB.^24^ Impaired BRB integrity exacerbates neurovascular injury and, if left untreated, leads to irreversible visual impairment.^25^ Notably, inhibition of ferroptosis in diabetic and oxidative injury models has been shown to attenuate RPE damage, preserve retinal structure, and delay disease progression,^25^ highlighting the pathological relevance of ferroptosis in DR. However, the upstream regulators and molecular mechanisms driving ferroptosis in RPE cells under diabetic stress remain poorly understood. Investigating these mechanisms is crucial for uncovering novel therapeutic targets aimed at preventing ferroptosis‐induced retinal degeneration in DR.

NR4A1 is broadly involved in the regulation of cell proliferation, apoptosis, inflammation, and metabolic responses in multiple tissues, and it can modulate gene expression by binding to specific response elements as a monomer or dimer on target promoters.^26^ NR4A1 has been implicated in various metabolic processes and stress responses.^27^ For example, its expression was increased in the retina of mice fed a high‐fat diet, where it promoted expression of genes involved in metabolic pathways,^14^ suggesting a role in retinal metabolic adaptation and dysregulation in diet‐associated disease. In the context of diabetes, NR4A1 modulated cellular glucose responses and was required for proper glucose‐stimulated insulin secretion in pancreatic β cells.^28^ Additionally, in DR, NR4A1 contributed to mitochondrial dysfunction and tissue damage.^29^ In this study, we expand the current understanding of NR4A1 in DR by demonstrating that NR4A1 knockdown significantly attenuates HG‐induced ferroptosis in ARPE‐19 cells. These findings identify NR4A1 as a key mediator of ferroptosis in RPE cells under diabetic conditions. Targeting NR4A1 may therefore represent a promising strategy to mitigate ferroptotic damage in DR.

Ubiquitination is a key post‐translational modification regulating protein stability and signaling.^30^ It involves E1, E2, and E3 enzymes, with E3 ubiquitin ligases conferring substrate specificity.^30^ SMURF2 is a well‐characterized E3 ligase that regulates various signaling pathways and protein turnover.^31^ Increasing evidence indicates that SMURF2 also plays a role in the pathogenesis of diabetes‐related diseases.^32^, ^33^, ^34^ For instance, Chen et al. demonstrated that SMURF2 regulated the progression of diabetic nephropathy by affecting mesangial cell proliferation and fibrosis.^32^ Additionally, miR‐195 has been shown to promote epithelial–mesenchymal transition and increase cell permeability in HG‐stimulated RPE cells by inhibiting SMURF2‐mediated ubiquitination and degradation of YY1.^6^ Building on these findings, our study identified NR4A1 as a novel substrate of SMURF2. We demonstrated that SMURF2 interacted with NR4A1 and promoted its ubiquitination and degradation, thereby limiting NR4A1‐mediated ferroptosis. SMURF2 overexpression alleviated HG‐induced ferroptosis in vitro and reduced ferroptosis‐associated markers in the retinas of diabetic mice. Notably, co‐overexpression of NR4A1 reversed the protective effects of SMURF2, underscoring a functional antagonism. Collectively, our results reveal a new mechanistic axis in which SMURF2 negatively regulates NR4A1 stability and ferroptosis, linking post‐translational regulation to the pathogenesis of DR.

FOXO6 is known to regulate stress responses, cell survival, and metabolic pathways. Accumulating evidence indicates that FOXO6 plays an important role in diabetes‐related pathophysiology, linking systemic metabolic dysregulation to tissue‐specific cellular injury. Previous studies have shown that FOXO6 contributed to oxidative damage and apoptosis in retinal cells under diabetic conditions.^19^ In parallel, FOXO6 depletion was reported to protect against diet‐induced glucose intolerance and insulin resistance in mice by attenuating hepatic gluconeogenesis and reducing macrophage infiltration in the liver and adipose tissues.^35^ However, whether FOXO6 modulates ferroptosis or interacts with ubiquitination machinery in DR has not been explored. Our study fills this gap by identifying SMURF2 as a direct transcriptional target of FOXO6 in RPE cells. We demonstrated that FOXO6 repressed SMURF2, thereby stabilizing NR4A1 and promoting ferroptosis. These findings not only extend previous knowledge of FOXO6's role in diabetic pathology but also uncover a new FOXO6–SMURF2–NR4A1 regulatory axis linking transcriptional repression to ubiquitin‐mediated ferroptosis in the DR.

In conclusion, this study identifies a novel regulatory axis in which FOXO6 transcriptionally represses SMURF2, leading to decreased ubiquitination and degradation of NR4A1 and consequently promoting ferroptosis in RPE cells under diabetic conditions. These findings advance our understanding of ferroptosis regulation in DR and suggest that SMURF2 may be a therapeutic target to limit NR4A1 accumulation and modulate ferroptotic signaling.

However, several limitations should be acknowledged. First, although both in vivo and in vitro models were employed, the study was limited to mice and the ARPE‐19 cell line; validation in primary human RPE cells or patient‐derived retinal tissues is required to confirm translational relevance. Second, although FOXO6 has been shown to regulate oxidative stress and apoptosis in RPE cells, whether the FOXO6/SMURF2/NR4A1 axis also contributes to these processes, in addition to ferroptosis, remains to be determined. Given the overlap between oxidative stress, apoptosis, and ferroptosis in DR, this axis may integrate multiple forms of RPE injury. Future investigations addressing these gaps will be essential to fully define the role of the FOXO6/SMURF2/NR4A1 axis in DR and assess its potential as a therapeutic target.

## AUTHOR CONTRIBUTIONS

Qiao Chen designed this study. Bo Su and Ke Xu collected the materials and performed the experiments. Qiao Chen and Liang Li analyzed the data and wrote the manuscript. Changzheng Chen revised the manuscript. All authors read and approved the final version of the manuscript.

## CONFLICT OF INTEREST STATEMENT

These authors declared no competing interests in this work.

## ETHICS STATEMENT

All animal procedures complied with the Guide for the Care and Use of Laboratory Animals and were approved by the Ethics Committee of Health Science Genter, Yangtze University.

## Supporting information

Supporting Information S1

## Data Availability

The raw data supporting the conclusions of this manuscript will be made available by the corresponding author, without undue reservation, to any qualified researcher.
