# Supplementary material for: FOXO6 inhibits SMURF2‐mediated ubiquitination and degradation of NR4A1 to promote retinal pigment epithelial cell ferroptosis in diabetic retinopathy
Source: J Cell Commun Signal. 2026 Aug 26;20(3):e70108. doi: 10.1002/ccs3.70108 (PMC13507828; doi:10.1002/ccs3.70108)
Supplement: Supplementary file 1 [file CCS3-20-e70108-s001.docx]

**Supplementary Figures and Figure Legends**

**Supplementary Figure 1. Bioinformatic prediction and validation of NR4A1-interacting ubiquitin regulators**

(A, B) Protein interaction prediction for NR4A1 using the BioGRID database. (C, D) Co-IP assays performed in ARPE-19 cells to assess protein–protein interactions between NR4A1 and OTUB1 (C), or OTUB2 (D). *n* = 3. Data are presented as mean ± SD.

**Supplementary Figure 2. The co-localization of SMURF2 and NR4A1 in the RPE layer of diabetic mice**

Representative immunofluorescence images showing SMURF2 and NR4A1 expression and localization in the RPE layer of male C57BL/6 mice from the Control, DM, DM + oe-NC, DM + oe-SMURF2, and DM + oe-SMURF2 + oe-NR4A1 groups. DAPI was used for nuclear staining. Scale bar, 20 μm.

**Supplementary Figure 3. Detection of protein interactions between FOXO6 and SMURF2 in ARPE-19 cells**

Co-IP analysis of the potential interaction between SMURF2 and FOXO6 in ARPE-19 cells.
